# Comparing Reentrant Drivers Predicted by Image-Based Computational Modeling and Mapped by Electrocardiographic Imaging in Persistent Atrial Fibrillation

**DOI:** 10.3389/fphys.2018.00414

**Published:** 2018-04-19

**Authors:** Patrick M. Boyle, Joe B. Hakim, Sohail Zahid, William H. Franceschi, Michael J. Murphy, Edward J. Vigmond, Rémi Dubois, Michel Haïssaguerre, Mélèze Hocini, Pierre Jaïs, Natalia A. Trayanova, Hubert Cochet

**Affiliations:** ^1^Department of Biomedical Engineering, Institute for Computational Medicine, Johns Hopkins University, Baltimore, MD, United States; ^2^L'Institut de RYthmologie et Modélisation Cardiaque (IHU-LIRYC), Pessac-Bordeaux, France; ^3^Centre Hospitalier Universitaire de Bordeaux, Pessac-Bordeaux, France

**Keywords:** atrial fibrillation, reentrant drivers, fibrotic remodeling, ablation, computational modeling, electrocardiographic mapping

## Abstract

Electrocardiographic mapping (ECGI) detects reentrant drivers (RDs) that perpetuate arrhythmia in persistent AF (PsAF). Patient-specific computational models derived from late gadolinium-enhanced magnetic resonance imaging (LGE-MRI) identify all latent sites in the fibrotic substrate that could potentially sustain RDs, not just those manifested during mapped AF. The objective of this study was to compare RDs from simulations and ECGI (RD_sim_/RD_ECGI_) and analyze implications for ablation. We considered 12 PsAF patients who underwent RD_ECGI_ ablation. For the same cohort, we simulated AF and identified RD_sim_ sites in patient-specific models with geometry and fibrosis distribution from pre-ablation LGE-MRI. RD_sim_- and RD_ECGI_-harboring regions were compared, and the extent of agreement between macroscopic locations of RDs identified by simulations and ECGI was assessed. Effects of ablating RD_ECGI_/RD_sim_ were analyzed. RD_sim_ were predicted in 28 atrial regions (median [inter-quartile range (IQR)] = 3.0 [1.0; 3.0] per model). ECGI detected 42 RD_ECGI_-harboring regions (4.0 [2.0; 5.0] per patient). The number of regions with RD_sim_ and RD_ECGI_ per individual was not significantly correlated (*R* = 0.46, *P* = ns). The overall rate of regional agreement was fair (modified Cohen's κ_0_ statistic = 0.11), as expected, based on the different mechanistic underpinning of RD_sim_- and RD_ECGI_. nineteen regions were found to harbor both RD_sim_ and RD_ECGI_, suggesting that a subset of clinically observed RDs was fibrosis-mediated. The most frequent source of differences (23/32 regions) between the two modalities was the presence of RD_ECGI_ perpetuated by mechanisms other than the fibrotic substrate. In 6/12 patients, there was at least one region where a latent RD was observed in simulations but was not manifested during clinical mapping. Ablation of fibrosis-mediated RD_ECGI_ (i.e., targets in regions that also harbored RD_sim_) trended toward a higher rate of positive response compared to ablation of other RD_ECGI_ targets (57 vs. 41%, *P* = ns). Our analysis suggests that RDs in human PsAF are at least partially fibrosis-mediated. Substrate-based ablation combining simulations with ECGI could improve outcomes.

**Graphical Abstract:**
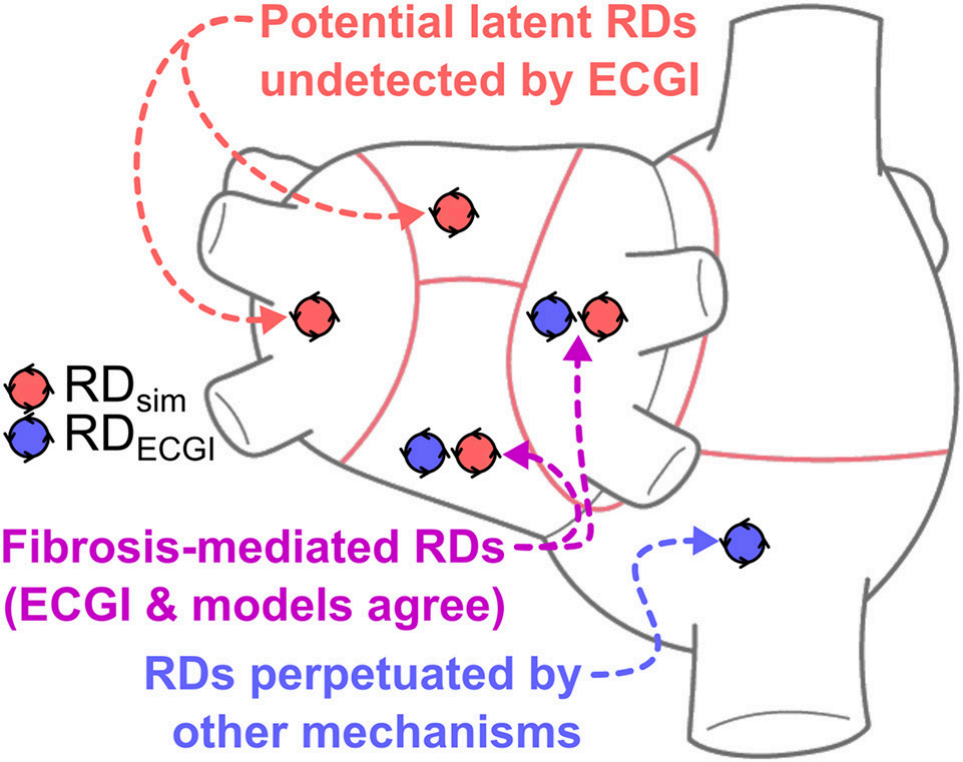
This study presents a comparison between reentrant driver (RD)-harboring regions identified by electrocardiographic imaging (ECGI), conducted prior to catheter ablation in persistent atrial fibrillation (PsAF) patients, and via simulations conducted in patient-specific computational models reconstructed from late gadolinium-enhanced magnetic resonance imaging (LGE-MRI) scans. The finding of atrial regions in which both ECGI and simulations detected RDs (purple) suggests that PsAF is at least partially driven by fibrosis-mediated mechanisms. Simulations also identify “latent” RDs (red)—regions within the fibrotic substrate where an RD could persist, but never manifested during clinical mapping. Conversely, RD-harboring regions identified by ECGI but not in simulations (blue) indicate that some clinically mapped AF episodes were perpetuated by mechanisms other than the fibrotic substrate. Our retrospective analysis suggests that substrate-based ablation combining simulations with ECGI could improve outcomes.

## Introduction

Atrial fibrillation (AF) is the most prevalent sustained arrhythmia, affecting 1–2% of the population (Andrade et al., 2014). Catheter ablation to isolate the pulmonary veins (PVs) has emerged as an effective treatment for some forms of AF (Haïssaguerre et al., 1998; Calkins et al., 2017), but outcomes are poor in patients with persistent AF (PsAF), with recurrence rates of 40–60% (Verma et al., 2015). The primary impediment to effective PsAF ablation is the presence of significant atrial remodeling (Burstein and Nattel, 2008; Yue et al., 2011), the arrhythmogenic propensity of which cannot be eliminated by PV isolation. Thus, there is an urgent need for new approaches that can result in accurate identification of optimal ablation targets for PsAF.

A promising non-invasive approach uses electrocardiographic mapping (ECGI) to reconstruct atrial activations from body surface potentials and approximate the locations of reentrant drivers (RDs; i.e., rotors) sustaining AF, which are targeted for ablation (Haissaguerre et al., 2014; Lim et al., 2017). Although initial studies show that patient outcomes after ECGI-driven ablation are promising, AF still recurs within one year in 20% of patients who receive ECGI-driven RD ablation (Haissaguerre et al., 2014). In cases of failed ablation, AF is typically sustained by persistent RDs at different locations (Lalani et al., 2016). This indicates that ECGI-driven RD ablation did not modify the arrhythmogenic substrate sufficiently to eliminate its capacity tosustain RDs and thus achieve freedom from AF. Thus, there is a need to augment observations from ECGI with information from additional sources.

Fibrosis is a major component of pro-AF remodeling (Nattel et al., 2008). Recently, our team developed a new methodology for simulating fibrosis-mediated AF in personalized atrial models reconstructed from LGE-MRI (McDowell et al., 2012; Boyle et al., 2016; Zahid et al., 2016a). This approach was used to identify patient-specific characteristics of the fibrotic tissue spatial distribution that promote AF (Zahid et al., 2016a). Specifically, we showed that RDs dynamically localize and anchor to boundary zones between fibrotic and non-fibrotic tissue with high fibrosis density and entropy. Clinical evidence in support of this finding has subsequently emerged (Cochet et al., 2018). Because each individual model can be sequentially paced from dozens of atrial locations to induce AF, simulations can pinpoint all locations within the fibrotic substrate capable of sustaining latent RDs. These are defined as regions within the fibrotic substrate where an RD could persist, but never manifested during clinical mapping. Thus, ablation at targets predicted by simulations conducted in image-based models has the potential to fully eliminate the arrhythmogenic capacity of the fibrotic substrate.

The aim of this study was to contrast the ablation target predictions by these two non-invasive strategies, ECGI and image-based computational modeling, in patients with AF and fibrosis, and to compare acute results of clinical RD_ECGI_ ablation with retrospective ablation of RD_sim_ in the same patients. This comparison offers insights into the presence of latent RDs in the fibrotic atria, which have important implications for improving the clinical procedure of PsAF ablation. Furthermore, since the image-based computational modeling methodology is based on the assessment of RDs driven exclusively by the fibrotic substrate, the comparison provides insights on whether RDs observed in a given clinical PsAF episode are fibrosis-mediated.

## Materials and methods

### Population

Twelve PsAF patients with fibrotic remodeling identified by LGE-MRI were retrospectively included in this study. This was a subset of a 20-patient cohort used in a previous study (Zahid et al., 2016a), which aimed to explore computationally how AF dynamics relate to the spatial characteristics of each individual's unique distribution of fibrotic tissue. This study was approved by the Institutional Ethics Committee at the University of Bordeaux, and all patients gave informed consent. This investigation conformed to the principles outlined in the Declaration of Helsinki. For the present follow-up study, which has distinct aims and scope (as described in the Introduction), we considered the subset of 12 cases in which at least one RD was identified by ECGI (RD_ECGI_) and at least one RD was induced in the corresponding patient-specific model (RD_sim_).

### Identification of RD_sim_ and RD_ECGI_

**Figure 1:**
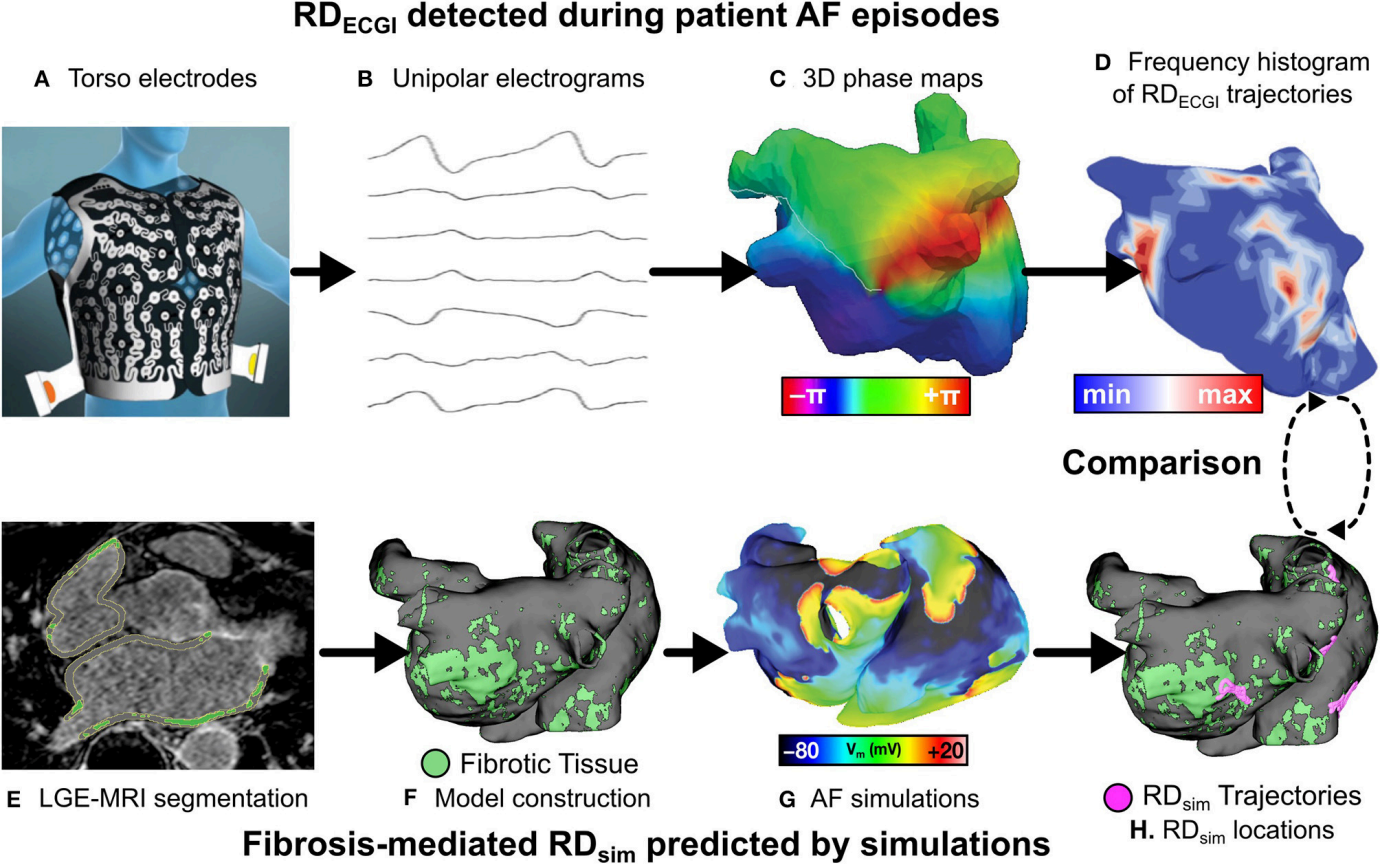
Workflow for comparison of RD_sim_ and RD_ECGI_ locations. Torso electrodes recorded 15 s of pre-ablation AF in PsAF patients **(A)**, and unipolar electrograms were reconstructed **(B)**. Phase maps **(C)** were analyzed to construct RD-phase singularity histograms **(D)**. Each patient underwent LGE-MRI **(E)**, which was used to reconstruct 3D atrial models **(F)**. Programmed electrical stimulation induced in-silico AF **(G)**, and fibrosis-driven RD-phase singularity trajectories in simulations were determined **(H)**. RDs from ECGI and simulations were compared. Panel **(A)** is reused with permission from Cochet et al. (2018).

All 12 patients underwent pre-ablation ECGI to identify regions in which RDs manifested during clinical AF episodes. The ECGI methodology is described in previous publications (Haissaguerre et al., 2013, 2014; Lim et al., 2017). Briefly, body surface potentials were acquired over 15s of AF with a 252-electrode vest (Figure 1A) (CardioInsight Technologies Inc., Cleveland, OH). Computed tomography was used to assess electrode locations with respect to bi-atrial geometry and epicardial unipolar electrograms were reconstructed (Figure 1B; Oster et al., 1998). Phase mapping was applied to visualize AF dynamics (Haissaguerre et al., 2013; Figure 1C) and identify phase singularities (i.e., organizing centers), which were displayed via histogram maps (Haissaguerre et al., 2014) to identify RD_ECGI_ (i.e., regions of local maxima in Figure 1D).

Reconstruction of patient-specific atrial models for the same 12 patients was described previously (McDowell et al., 2012; Zahid et al., 2016a,b; Deng et al., 2017). The atrial wall was segmented from MRI scans and LGE and non-LGE regions were segmented using an adaptive histogram thresholding algorithm (Jadidi et al., 2013). As shown previously Jadidi et al. (2013), LGE intensity within each slice follows a bi-modal distribution (i.e., distinct peaks associated with LGE and non-LGE regions). The intensity threshold that best separated these voxel populations was chosen by an operator who was blind to the results of the study (Zahid et al., 2016a). Three-dimensional finite-element meshes were then constructed for each patient-specific model (Figure 1F; McDowell et al., 2012; Zahid et al., 2016a). These models included a realistic representation of finite atrial wall thickness, with inter- and intra-patient variability thereof. Average edge length ranged from 462 to 468 μm.

Myocyte membrane dynamics in non-fibrotic regions were represented with a human atrial model under chronic AF conditions (Courtemanche et al., 1998; Krummen et al., 2012). In fibrotic regions, additional changes described by Zahid et al. (Roney et al., 2016; Zahid et al., 2016a) were made to represent changes due to fibrogenic remodeling: −50% I_K1_, −50% I_CaL_, −40% I_Na_. Finally, as described previously, realistic atrial fiber orientations were mapped into each patient-specific model from a human atlas of atrial geometry (Krueger et al., 2011) and anisotropic conduction velocities in fibrotic and non-fibrotic regions were calibrated to match the range of known clinical values (Zahid et al., 2016a). In each model, rapid pacing was applied at 30 evenly-distributed sites to induce AF (Figure 1G; Zahid et al., 2016a). Each induced AF episode was simulated for 2.5 s following the end of pacing. Phase singularity trajectories were tracked over time using spatiotemporal clustering to identify RD_sim_, defined as spatially-confined trajectories persisting ≥2 rotations and ≥200 ms (Figure 1H; Zahid et al., 2016a). This definition was consistent with criteria used to define RDs by ECGI (Narayan et al., 2012; Haissaguerre et al., 2014). Notably, our definition of RD activity specifically excludes macro-reentrant activity around non-conductive obstacles (e.g., atrial “flutter” around the mitral or tricuspid valve annulus).

### Comparison of RD_sim_- and RD_ECGI_-harboring regions

**Figure 2:**
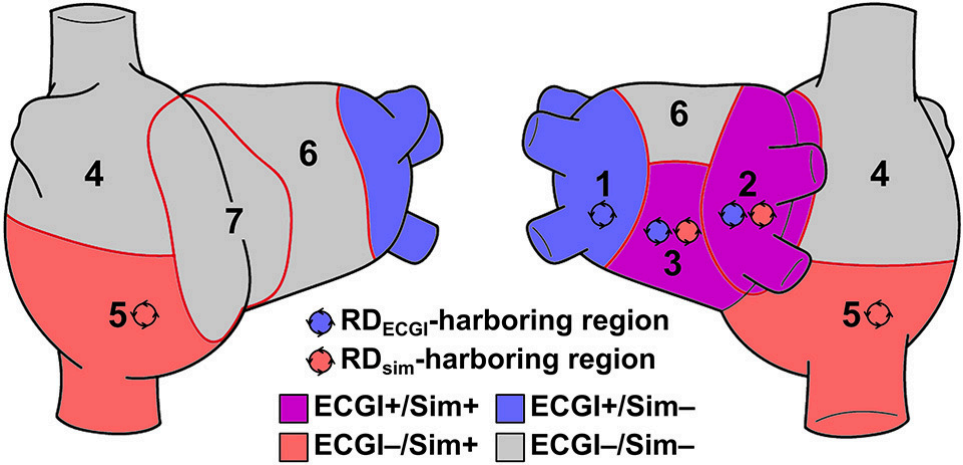
Schematic defining region classification. RD_ECGI_ and RD_sim_ were classified into atrial regions as follows: (1/2) left/right PVs; (3) posterior LA; (4/5) superior/inferior RA; (6) anterior LA; and (7) inter-atrial groove. RD_ECGI_- and RD_sim_-harboring regions in this schematic are for illustrative purposes only and are not related to any particular patient/model.

We compared RD_sim_ and RD_ECGI_ locations on a region-wise basis. Each atrial geometry was subdivided into seven anatomically-defined regions, as described by Haissaguerre et al. (2014): four regions in the left atrium (LA), two in the right atrium (RA), and one in the interatrial septum. Each RD was classified as belonging to the region in which the majority of its phase singularities persisted. For each patient, regions in which both RD_sim_ and RD_ECGI_ were observed, were categorized as ECGI+/Sim+; following the same convention, other regions were categorized as ECGI+/Sim−, ECGI−/Sim+, or ECGI−/Sim−. Individuals who categorized RD_ECGI_ locations were independent and blinded from those who conducted simulations and classified RD_sim_ locations. Figure 2 presents a schematic representation of the RD classification approach, including an illustration of the seven atrial regions.

It is important to note that while RD_ECGI_ sites estimate the locations of AF-perpetuating sources driving the current clinical episode in each patient, RD_sim_ represent *all* sites within each patient's individual fibrotic substrate where rotors *could potentially* be sustained. As such, we expect only partial agreement between RD_ECGI_ and RD_sim_. This suggests that RD locations of agreement between ECGI and image-based computational modeling are RDs maintained by the fibrotic substrate during clinical AF episodes, while RDs detected in ECGI only are indicative of AF-sustaining mechanisms that are not fibrosis related.

### Clinical ablation of RD_ECGI_ and simulated ablation of RD_sim_

Ablation of RD_ECGI_ sites was performed in patients as part of a previous study (Haissaguerre et al., 2014). Since acute outcomes of these procedures are included in our results and correlated with RD activity observed in corresponding patient-specific models, we provide here a brief summary of how ablation was conducted. ECGI-driven ablation was performed in regions exhibiting high RD activity. The endpoint of regional ablation was local electrogram slowing and organization (i.e., conversion of local rapid complex signals into slower simple signals). As in previous studies, positive response to ablation was defined by acute termination or cycle length prolongation >10 ms. Acute success or failure of RD ablation, as reported in the present study, was assessed prior to performing pulmonary vein isolation at the conclusion of each clinical procedure.

Ablation was also simulated in each computational model, in each case targeting all RD_sim_ sites. This is the first study in which *in-silico* ablation and subsequent AF inducibility studies were conducted for this patient cohort. Ablation was modeled by rendering tissue along RD phase singularity trajectories non-excitable (3.5 mm lesion radius). We then repeated the multi-site pacing protocol and, in cases where AF could still be induced post-ablation (i.e., perpetuated by emergent reentrant drivers), regions in which *de novo* RD_sim_ occurred were noted. We could not simulate ablation of observed RD_ECGI_ locations because, while some were annotated using a clinical mapping system, others were logged qualitatively in case reports. Consistent with the definition provided above, macro-reentrant activity propagating around virtual simulated ablation lesions (i.e., around a region of non-conductive tissue and lacking a trajectory of dynamically meandering phase singularities) was explicitly not classified as RD_sim_.

### Statistics

Continuous variables were expressed as median [IQR] and compared using the Wilcoxon signed-rank test. Categorical variables were expressed as percentages and compared using Fisher's exact test. After classifying RD_ECGI_ and RD_sim_ within anatomical regions (see Figure 2), inter-rater agreement between RD_ECGI_ and RD_sim_ locations was assessed by calculating the modified Cohen's kappa statistic (κ_0_) (Kraemer, 1980). All tests were two-tailed; *P* < 0.05 indicated statistical significance.

## Results

### RDs identified by both modalities

**Figure 3:**
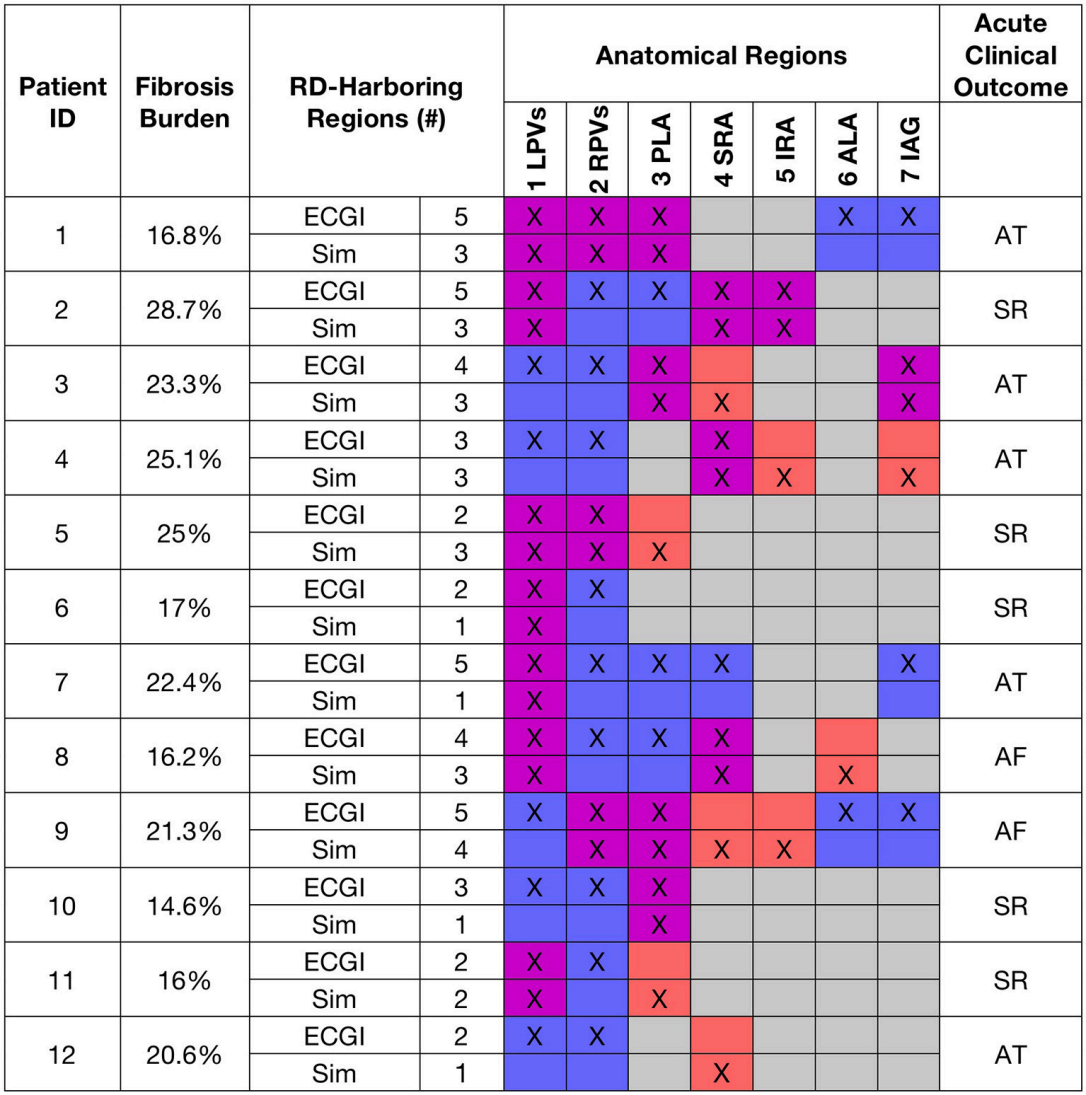
Summary of RD-related findings for all patients and corresponding models. Table shows fibrosis burden, number and distribution of RD_sim_- and RD_ECGI_-harboring atrial regions, and acute clinical outcome of ECGI-driven ablation, i.e., continued AF or termination to sinus rhythm (SR) or atrial tachycardia (AT). Cell pairs are color-coded using the same scheme as Figure 2.

Figure 3 shows fibrosis burden, anatomical regions where RD_sim_ and RD_ECGI_ were detected, and acute ECGI-driven ablation outcomes for all patients. RD_ECGI_ were detected in 42 atrial regions (4.0 [2.0; 5.0] per patient). The distribution of RD_ECGI_-harboring regions was as follows: 33 (78.6%) in the LA, 5 in the RA (11.9%), and 4 (9.5%) in the inter-atrial groove. Within the LA, RD_ECGI_ were most frequently observed in the left and right PV regions (29% in each). RD_sim_ were detected in a total of 28 regions (3.0 [1.0; 3.0] per model). The distribution of RD_sim_-harboring regions was as follows: 17 (60.7%) in the LA, 9 (32.1%) in the RA, and 2 (7.1%) in the inter-atrial groove. Two thirds of all RD_sim_ observed were in three regions: the left PV (25.0%), posterior LA (21.4%), and the superior RA (21.4%).

**Figure 4:**
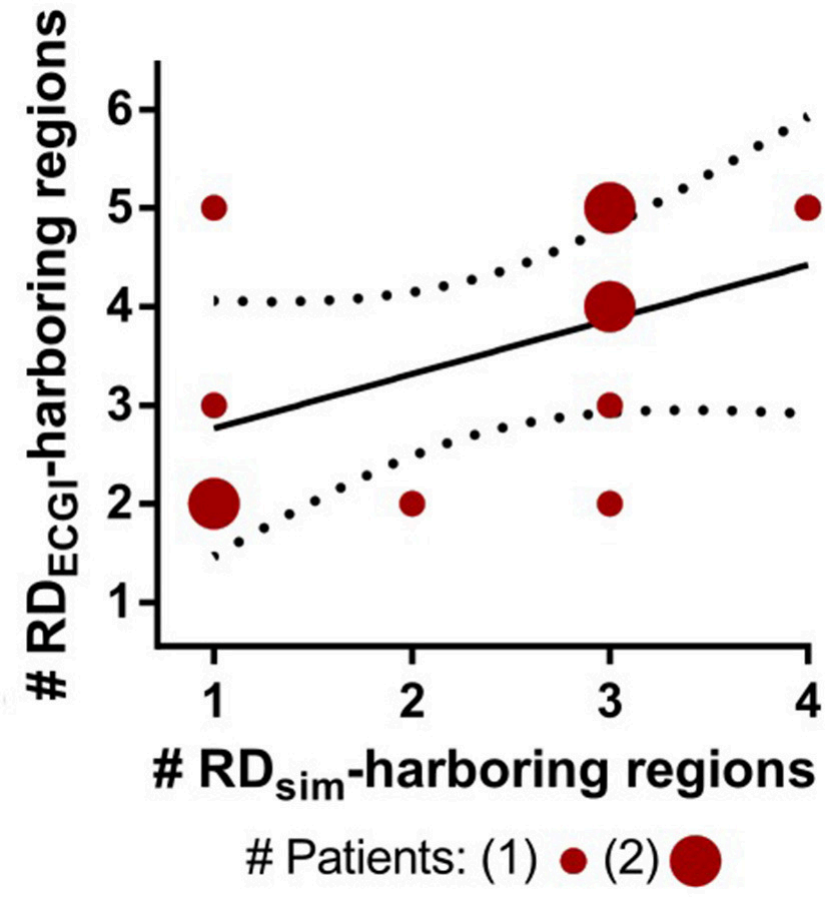
Relationship between the numbers of RD_sim_-harboring regions predicted by each model and RD_ECGI_-harboring regions observed during clinically mapped AF in the corresponding patients. Solid and dotted lines indicate best fit for linear regression and 95% confidence intervals, respectively.

**Figure 5:**
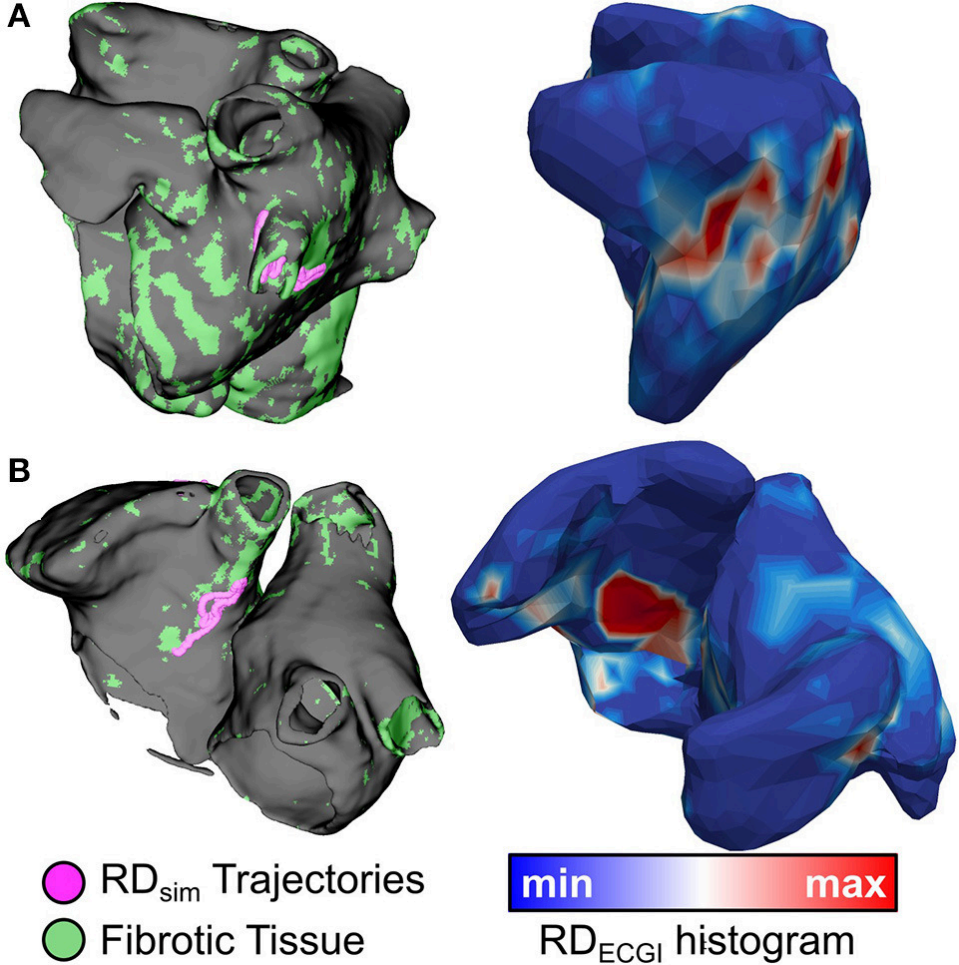
Spatial co-localization of RD_sim_ (left) and RD_ECGI_ (right). **(A)** (patient 7): Matching RD_sim_ and RD_ECGI_ sites in the left PV region. **(B)** (patient 3): Matching RD_sim_ and RD_ECGI_ sites in the inter-atrial groove region.

For patients with a larger number of RD_ECGI_-harboring regions, there was a similar trend toward more RD_sim_-harboring regions in the corresponding models (Figure 4), but correlation was not statistically significant (*R* = 0.46, *P* = ns). ECGI and simulations agreed on classification of atrial regions (as RD-harboring or not) in a narrow majority of cases (5.0 [3.3; 5.0] vs. 3.0 [2.0; 3.8], see Table 1). Quantitative analysis of inter-rater agreement yielded κ_0_ = 0.11, which indicates a fair degree of consensus (Kraemer, 1980). These observations are consistent with our expectation that RD_sim_ and RD_ECGI_ locations would only partially agree, as described above, because of the partial overlap of mechanisms underpinning these RDS. Co-localization between RD_sim_ and RD_ECGI_ is illustrated for three patients in Figure 5.

**Table 1:** For each patient model, number of atrial regions in which ECGI and Simulations agreed (i.e., ECGI+/Sim+ or ECGI−/Sim−) and differed (i.e., ECGI+/Sim− or ECGI−/Sim+).

**Patient ID**	**Number of ECGI+/Sim+ & ECGI−/Sim− Regions**	**Number of ECGI+/Sim− & ECGI−/Sim+ Regions**
1	5	2
2	5	2
3	4	3
4	3	4
5	6	1
6	6	1
7	3	4
8	4	3
9	2	5
10	5	2
11	5	2
12	4	3

**Figure 6:**
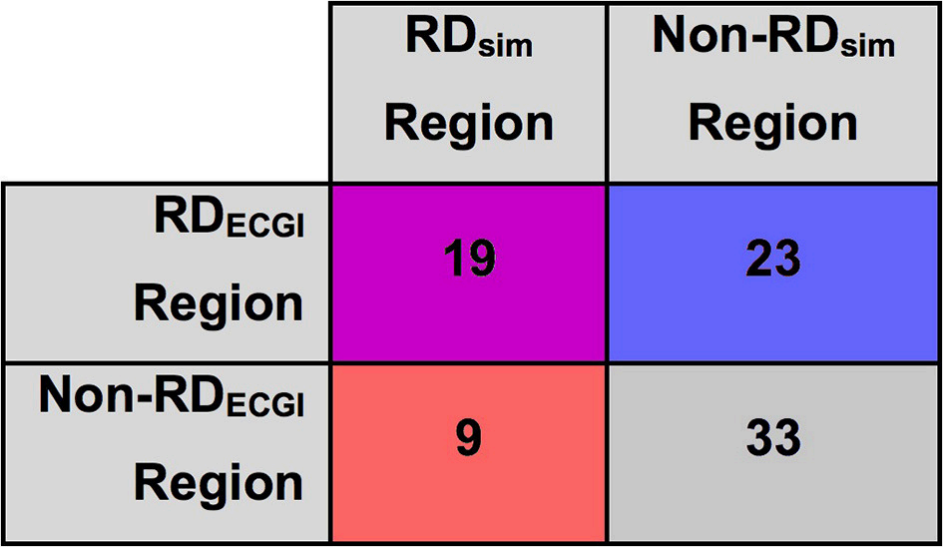
Inter-rater agreement between RD_sim_ and RD_ECGI_ regions in patient-derived models. Cells color-coded via same classification scheme used in Figure 3.

There were 19 regions where RDs were found by both approaches (ECGI+/Sim+), 33 regions where RDs were found by neither approach (ECGI−/Sim−), 23 regions where ECGI detected an RD while simulations did not (ECGI+/Sim−), and 9 regions where simulations identified an RD while ECGI did not (ECGI−/Sim+) (Figure 6).

The co-localization of RD_ECGI_ and RD_sim_ (or lack thereof) has important implications for understanding PsAF mechanisms. For regions classified as ECGI+/Sim+, our findings suggest that the clinically-observed rotor may have been perpetuated by the fibrotic substrate, since at least one rotor was induced in a similar location in simulations. Additionally, ECGI−/Sim+ regions suggest the presence of areas in the fibrotic substrate where the potential to induce an RD exists, even though rotor activity did not manifest (or was not observed) during clinically mapped AF episodes. Finally, activity in ECGI+/Sim− regions indicates the presence of AF perpetuation mechanisms other than fibrotic remodeling, which are not accounted for in our simulations.

**Figure 7:**
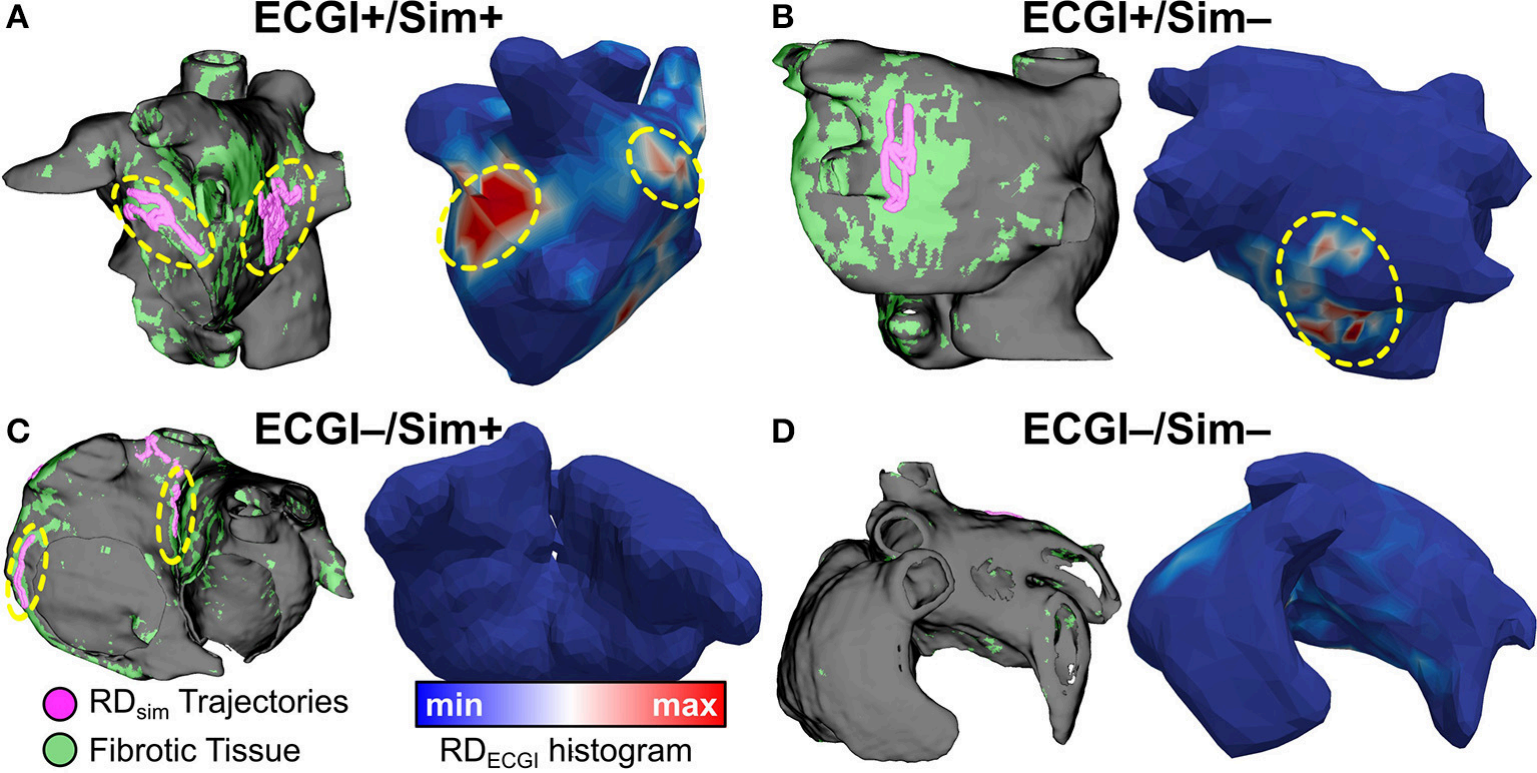
ECGI+/Sim+, ECGI+/Sim−, ECGI−/Sim+, and ECGI−/Sim− region examples. RD trajectories in regions of interest for each panel are highlighted by dashed yellow circles. **(A)** (patient 1): matching RD sites in both left PV and posterior LA regions. **(B)** (patient 2): RD_ECGI_ site in posterior LA region was not observed during simulations. **(C)** (patient 4): RD_sim_ sites in inferior RA and inter-atrial groove were not observed during clinical mapping via ECGI. **(D)** (patient 11): Superior RA and anterior LA regions were free of RD activity in both simulations and ECGI. See Figure 5 for legend.

**Figure 8:**
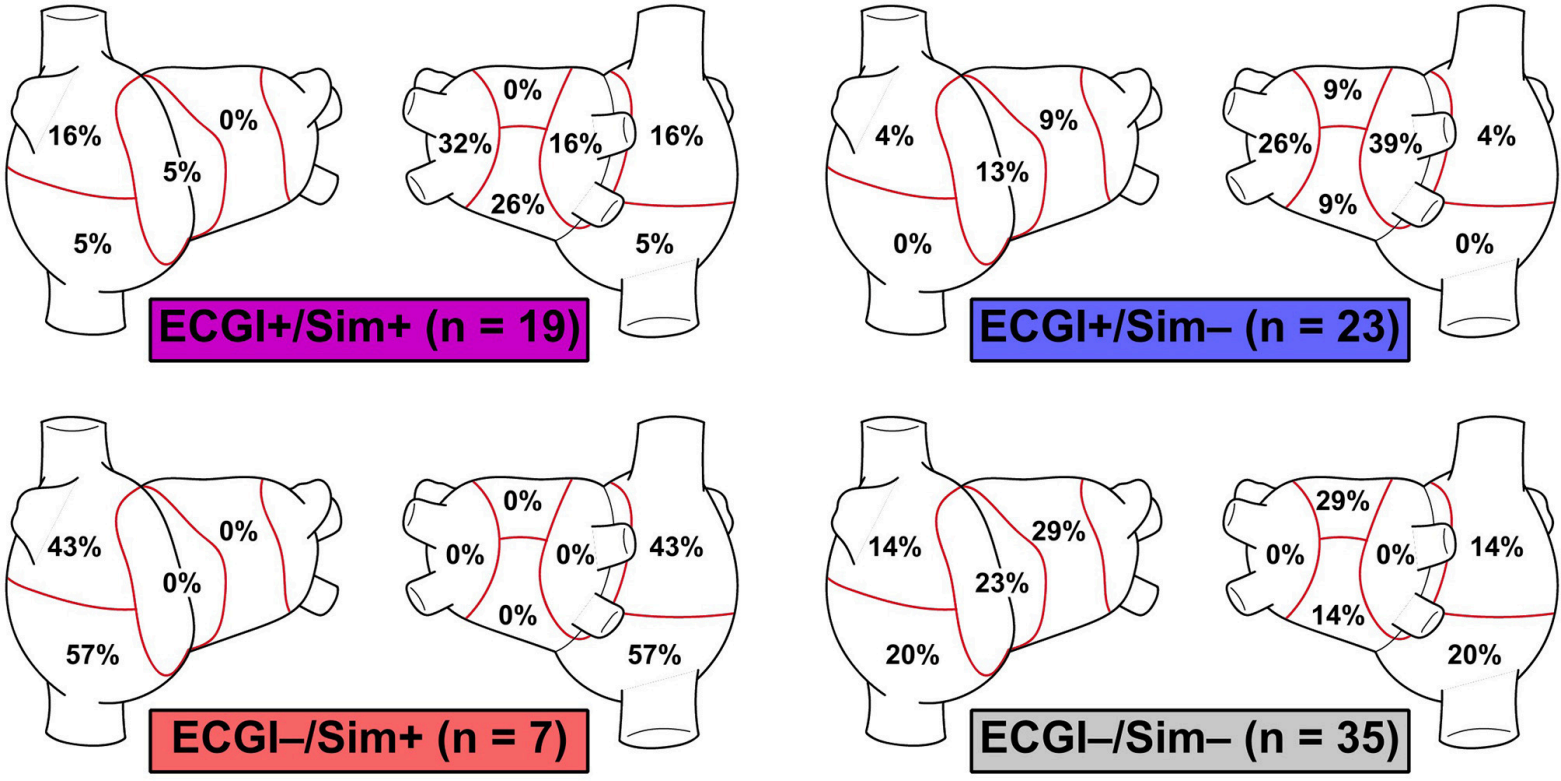
Regional distribution of ECGI+/Sim+, ECGI+/Sim-, ECGI-/Sim+ and ECGI-/Sim- regions. Each percentage value in each atrial region represents the proportion of all examples of the corresponding region classification (ECGI+/Sim+, etc.) corresponded to that region.

Side-by-side visualizations of RD_sim_ and RD_ECGI_ sites for ECGI+/Sim+, ECGI+/Sim−, ECGI−/Sim+, and ECGI−/Sim− regions are shown in Figure 7. The overall distribution of the different region types is shown in Figure 8. The most frequent ECGI+/Sim+ regions were the left PVs (37%) and posterior LA (21%). The most frequent ECGI+/Sim− regions were the right (39%) and left PVs (22%). The most frequent ECGI−/Sim+ regions were the superior (33%) and inferior RA (22%) and the posterior LA (22%). Lastly, the most frequent ECGI−/Sim− regions were the anterior LA (27%) the inferior RA (27%).

### Acute outcomes of RD_ECGI_-driven ablation

**Figure 9:**
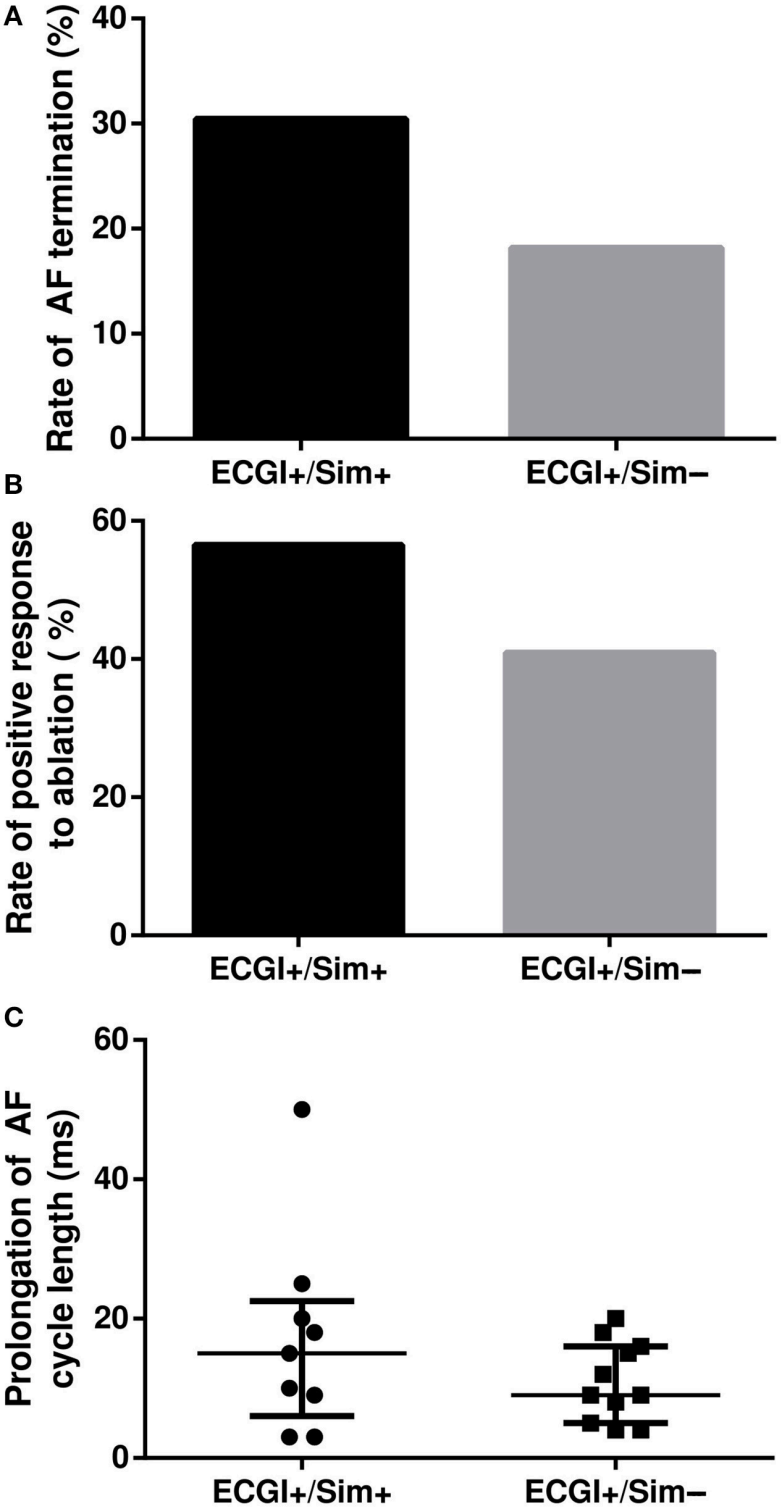
Outcome of RD_ECGI_ ablation in regions with and without RD_sim_. **(A,B)** Rates of AF termination and positive response to ablation in ECGI+/Sim− vs. ECGI+/Sim+ regions. **(C)** AF cycle length prolongation (median + upper/lower quartile lines) in response to ablation of RD_ECGI_ targets in ECGI+/Sim+ vs. ECGI+/Sim− regions.

For 45 distinct ECGI-determined targets ablated clinically, which were located in 42 atrial regions (23 ECGI+/Sim+, 22 ECGI+/Sim−), the average extent of ablated tissue was 6.8%. For each target, we retrospectively considered the acute ablation outcome. Interestingly, ablation of ECGI-predicted targets located in regions that were also RD_sim_-harboring (i.e., ECGI+/Sim+) led to higher rates of both AF termination [Figure 9A, 7/23 (30%) vs. 4/22 (18%), *P* = ns] and positive response to ablation [Figure 9B, 13/23 (57%) vs. 9/22 (41%), *P* = ns] compared to targets in ECGI+/Sim− regions. In cases where AF slowed but did not terminate, this also involved greater cycle length prolongation [Figure 9C, 15.0 [6.0; 22.5] ms (*n* = 9) vs. 9.0 [5.0; 16.0] ms, *P* = ns]. For the two patients in whom ECGI-driven RD ablation completely failed to terminate AF (i.e., rows labeled “AF” in Figure 3), simulations identified at least one potential latent RD that was not detected during ECGI (i.e., one or more ECGI−/Sim+ regions). In contrast, for 6/10 patients in whom ECGI-driven ablation converted AF to sinus rhythm or AT, there were no ECGI−/Sim+ regions (i.e., simulations did not uncover latent RDs).

### *In-silico* assessment of post-ablation AF inducibility

**Figure 10:**
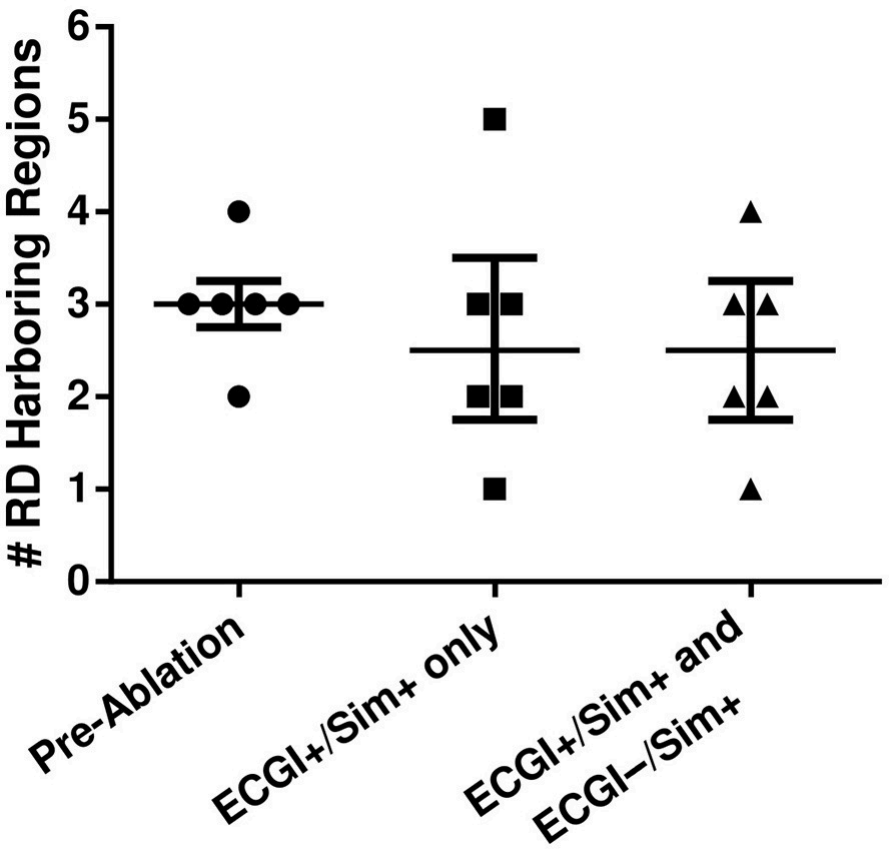
Number of RD_sim_-harboring regions in five patient-derived models before and after ablation of RD_sim_ targets. The first round of simulated ablation targeted sites within ECGI+/Sim+ regions only; the second additionally targeted RDs in ECGI−/Sim+ regions.

We simulated ablation and reassessed AF inducibility in the subset of six atrial models that had both ECGI+/Sim+ and ECGI−/Sim+ regions. For each model, two virtual ablation patterns were executed: first, RD_sim_ trajectories in ECGI+/Sim+ regions alone were ablated (3.3 [2.2; 4.5]% of atrial volume); second, RD_sim_ trajectories from both ECGI+/Sim+ and ECGI−/Sim+ regions were ablated (6.1 [5.2; 7.2]%). Outcomes are summarized in Figure 10 and Table 2. After the first set of ablations, AF remained inducible in all six models, although there was a trend toward reduction in the number of RD_sim_-harboring regions (3.0 [2.8; 3.3] to 2.5 [1.8; 3.5], *P* = ns). When the second lesion set was simulated, there was a slight further reduction in the number of RD_sim_-harboring regions (2.5 [1.8; 3.5] to 2.5 [1.8; 3.3], *P* = ns). Note that ablation of target(s) in a particular region did not necessarily preclude the initiation of new RDs in the same region in subsequent simulations, since only the RD trajectories, and not tissue around them, were ablated. We observed numerous cases (^*^ in Table 2) in which *de novo* RD_sim_ were induced in regions that were *not* RD_sim_-harboring in pre-ablation simulations. This suggests that potential RD sites existed in these regions pre-ablation but never manifested, possibly due to the fact that the distribution of fibrotic tissue in regions where RD_sim_ were observed had a very strong propensity for attracting reentrant activity. Interestingly, three of these *de novo* RD_sim_-harboring regions (^**^ in Table 2) were classified as ECGI+/Sim− in our analysis of pre-ablation simulations, suggesting an alternative explanation for some differences between RD_sim_ and RD_ECGI_ locations.

**Table 2:** Summary of number and distribution of RD_sim_ in pre-ablation models and after two rounds of *in-silico* ablation (1: RD_sim_ targets in ECGI+/Sim+ regions only; 2: all Sim+ targets).

**PID**	**RD-Harboring Regions (#)**		**Anatomical Regions**						
			**1 LPVs**	**2 RPVs**	**3 PLA**	**4 SRA**	**5 IRA**	**6 ALA**	**7 IAG**
3	ECGI	4	X	X	X				X
	Pre-ablation	3			X	X			X
	Post-Ablation 1	2				X	X		
	Post-Ablation 2	3	X^**^			X	X		
4	ECGI	3	X	X		X			
	Pre-ablation	3				X	X		X
	Post-Ablation 1	5	X^**^		X^*^	X	X		X
	Post-Ablation 2	4	X^**^		X^*^	X	X		
5	ECGI	2	X	X					
	Pre-ablation	3	X	X	X				
	Post-Ablation 1	3		X				X^*^	X^*^
	Post-Ablation 2	2		X				X^*^	
8	ECGI	4	X	X	X	X			
	Pre-ablation	3	X			X		X	
	Post-Ablation 1	1					X^*^		
	Post-Ablation 2	2					X^*^	X	
9	ECGI	5	X	X	X			X	X
	Pre-ablation	4		X	X	X	X		
	Post-Ablation 1	3		X		X	X		
	Post-Ablation 2	3	X	X			X		
11	ECGI	2	X	X					
	Pre-ablation	2	X		X				
	Post-Ablation 1	2	X		X				
	Post-Ablation 2	1	X						

*Asterisks indicate de novo Sim+ regions; double asterisks indicate regions that were classified as ECGI+/Sim− based on pre-ablation simulations*.

## Discussion

This study compared RDs predicted by image-based patient-specific computational models with RDs mapped clinically by ECGI in PsAF patients with fibrotic remodeling. The main findings are:

- There was weak but statistically non-trivial agreement between the regional distribution of RD_sim_ and RD_ECGI_. This was expected, as RD_sim_ represent all drivers that the fibrotic substrate could sustain, not just those manifested as RD_ECGI_ during mapping procedures.
- Partial co-localization of RD_sim_ and RD_ECGI_ suggests that human PsAF is at least partially driven by fibrosis-mediated RDs.
- ECGI-driven ablation was more likely to result in a positive outcome if the targeted RD_ECGI_ was in a region that is also found to be RD_sim_-harboring.
- In the two cases where ECGI-driven RD ablation failed to terminate AF, RD_sim_ were observed in regions where RD_ECGI_ were never mapped, suggesting that latent sites may be responsible for poor outcomes in some cases. In 6/10 acutely successful ablation procedures, no such latent sites were observed.
- *In-silico* ablation of RD_sim_ trajectories in ECGI+/Sim+ and ECGI+/Sim− regions was more effective than ablation of targets in ECGI+/Sim+ regions alone. As such, targeting regions in the fibrotic substrate associated with latent RDs may improve outcomes.

These results suggest that patient-derived modeling could be combined with ECGI to improve ablation and demonstrate that fibrotic remodeling plays a role in PsAF maintenance.

### Co-localization of RD_ECGI_ and RD_sim_

In both ECGI and simulations, a large proportion of RDs were located in the left PV and posterior LA regions (45.2 and 46.4%, respectively). These were also the two most frequently observed ECGI+/Sim+ regions [left PV: 7/19 (37%), posterior LA: 4/19 (21%)]. Since all RD_sim_ are inherently sustained by fibrotic remodeling, our simulations suggest that RD_ECGI_ located the ECGI+/Sim+ are also fibrosis-driven. This is consistent with previous studies, which showed that fibrotic tissue is most predominant, wavebreak is common, and reentrant circuits persist most frequently in these two regions (Tanaka et al., 2007).

Among all 32 atrial regions in which the presence of RD_sim_ and RD_ECGI_ differed, the majority (23/32) were ECGI+/Sim−. We surmise that the majority of RD_ECGI_ seen in such regions may be sustained by pro-arrhythmogenic mechanisms unrelated to the fibrotic substrate. Experiments conducted in ex-*vivo* hearts have shown that AF can be driven by reentrant circuits sustained by abrupt changes in wall thickness, micro-fibrosis, and sharp fiber angle variation (Hansen et al., 2015). Such micro-structural features cannot be visualized by current LGE-MRI and, accordingly, could not be incorporated in our models. Electrophysiological heterogeneities near the PVs, which are not represented in our models, can also promote reentry (Arora et al., 2003), which may explain why these two regions accounted for 61% of ECGI+/Sim− regions.

An additional source of differences between RD_sim_- and RD_ECGI_-harboring regions, which is supported by outcomes of virtual ablation experiments summarized in Table 2, is that some parts of the fibrotic substrate had such a strong propensity for attracting RDs that reentrant activity never had the chance to anchor at other potential locations (i.e., within ECGI+/Sim− regions). This stresses the point that it may be necessary to perform multiple rounds of simulations in each patient-specific model (i.e., attempt to induce AF, ablate RD targets, repeat) in order to derive an optimal set of targets in the fibrotic substrate that might sustain RDs.

As discussed in our previous study (Zahid et al., 2016a), LGE-MRI underestimates fibrotic tissue in certain areas (e.g., the LA roof). As such, we cannot exclude the possibility that some RD_ECGI_ observed in ECGI+/Sim− regions were actually fibrosis-mediated but could not be recapitulated in simulations due to intrinsic limitations of the imaging modality. Moreover, since ECGI maps activity on the atrial epicardium alone (Rudy, 2013), it is possible that the absence of clinically-observed RDs in some ECGI−/Sim+ regions might be explained by reentrant activity sustained in thicker parts of the atria such as the septum, which could be observed in models but not clinically. Finally, as explored in another recent study (Deng et al., 2017), there is intrinsic uncertainty in our atrial models with regards to the exact dynamics of RD localization. This uncertainty may also have contributed to the observed differences between RD_sim_- and RD_ECGI_-harboring regions seen in the present study.

### Implications for catheter ablation of RDs

Our discovery, in the retrospective analysis, that ablation of RD_ECGI_ in ECGI+/Sim+ regions had a positive effect more often than ablating targets in regions where RD_sim_ were not seen may have important implications. While statistical significance could not be achieved due likely to the small sample size, the finding nonetheless suggests that ablation may be more effective when it eliminates RDs sustained by the fibrotic substrate compared to those perpetuated by other mechanisms. Previous work has shown that anatomical reentry can arise when rotors attach to nonconductive obstacles in tissue (e.g., lesions) (Lim et al., 2006). We have previously shown that RDs dynamically localize to boundary regions between fibrotic and non-fibrotic tissue (Zahid et al., 2016a). Thus, we surmise that the likelihood of rotor attachment (i.e., which would manifest clinically as conversion from AF to macroscopic AT) is dramatically higher following ablation of RDs sustained by the fibrotic substrate. Identifying RD sites detected by both ECGI and simulation and treating them as priority targets may lead to earlier AF termination and prevent excessive ablation.

For the two patients in whom ECGI-driven RD ablation completely failed to terminate AF, simulations identified RD sites that were not detected by ECGI; these sites may have perpetuated AF post-ablation. Indeed, studies indicate that post-ablation AF can be sustained by RDs that occur in different locations than pre-ablation RDs (Lalani et al., 2016). This is also consistent with our finding in this study that ablating RD trajectories in ECGI−/Sim+ regions diminishes the capacity of the substrate to sustain AF. The implication is that ECGI-driven ablation [or other approaches based on RD identification, such as FIRM; Narayan et al., 2012) could be augmented by using simulations to identify additional fibrosis-mediated RDs to devise a more comprehensive ablation strategy. Further research involving prospective clinical studies with a greater number of patients is needed to assess the utility of prioritizing ablation of ECGI+/Sim+ regions and ablating ECGI−/Sim+ regions.

### Limitations

ECGI does not establish a ground truth representation for the dynamics of electrical activation in each individual's heart, but rather provides an estimate of the underlying phenomena. The accuracy of RD identification via ECGI has not been explicitly characterized. However, the reported accuracy for identifying the first sites of activation is ~4–6 mm (Cuculich et al., 2010; Wang et al., 2011; Haissaguerre et al., 2014); characterizing more complex activity (such as RDs) may be subject a higher degree of uncertainty. Moreover, ECGI is intrinsically limited to reconstructing activity on the epicardial surface of the heart (Rudy, 2013) and cannot directly visualize excitations on the inter-atrial septum (Haissaguerre et al., 2014). As such, reentrant activity that in these locations may be misclassified as an apparent focal source or be missed altogether.

Likewise, some limitations of image-based computational modeling are relevant to the process of RD_sim_ identification. LGE-MRI cannot identify regions of fibrotic remodeling in certain parts of the heart, such as the LA roof (Zahid et al., 2016a), so some potential RD anchoring sites may be overlooked. Moreover, to avoid the need to obtain invasive electrophysiological measurements (e.g., APD, CV) from each individual patient, our models assume average human AF electrophysiology at the cell- and tissue-scale. Although this approach dramatically expands the feasibility of conducting large computational modeling-based studies, we have shown previously that it also introduces uncertainty in the dynamics of RD localization (Deng et al., 2017).

## Conclusions

Our analysis reveals limited agreement between ECGI and patient-specific simulations based on LGE-MRI scans, as the two methodologies represent different aspects of the arrhythogenic propensity of the fibrotic substrate in PsAF patients. The presence of numerous atrial regions in multiple patients that were found to be RD-harboring by both ECGI and simulations suggests that PsAF is in part driven by fibrosis-mediated mechanisms. Ablation of RD_ECGI_ in regions where simulations indicate the substrate for RD_sim_ initiation exists compared to those in which it does not. Since simulations can identify latent RDs that may not manifest during clinical mapping, a substrate-based approach combining modeling and ECGI may improve long-term outcomes.

## Author contributions

PB, SZ, NT, and HC conceived the idea; PB, JH, SZ, WF, and MM developed the patient-specific atrial models, conducted computational simulations, and analyzed outputs thereof; RD, PJ, and HC obtained late gadolinium enhanced magnetic resonance images for each patient; PB, JH, SZ, EV, RD, MiH, MéH, PJ, NT, and HC interpreted imaging data and simulation results; PB, SZ, NT, and HC wrote the manuscript.

### Conflict of interest statement

MiH, MéH, and PJ are stockholders in CardioInsight Inc. RD is a paid consultant to CardioInsight Inc. EV and NT are cofounders of CardioSolv, LLC. CardioInsight and CardioSolv were not involved in this research. All other authors declare that the research was conducted in the absence of any commercial or financial relationships that could be construed as a potential conflict of interest.

## Abbreviations

AT: Atrial Tachycardia
LGE-MRI: Late Gadolinium-Enhanced Magnetic Resonance Imaging
LA: Left Atrium
PsAF: Persistent Atrial Fibrillation
PV: Pulmonary Vein
RA: Right Atrium
SR: Sinus Rhythm.
